# Effect of Ankaferd Blood Stopper on air leakage in the lung and prevention of bleeding: an experimental study

**DOI:** 10.1186/1749-8090-6-20

**Published:** 2011-02-27

**Authors:** Ali Kılıçgün, Necla G Sarıkaş, Tanzer Korkmaz, Özkan Saydam, Çetin Boran, Güledal Boztaş

**Affiliations:** 1 Department of Thoracic Surgery, School of Medicine, University of Abant İzzet Baysal, Bolu, Turkey; 2 Department of Pediatric Surgery, School of Medicine, University of Abant İzzet Baysal, Bolu, Turkey; 3 Department of Emergency, School of Medicine, University of Abant İzzet Baysal, Bolu, Turkey; 4 Department of Thoracic Surgery, School of Medicine, University of Karaelmas, Zonguldak, Turkey; 5 Department of pathology, School of Medicine, University of Abant İzzet Baysal, Bolu, Turkey; 6 Department of Public Healt, City Healt Administrative, Bolu, Turkey

## Abstract

**Background:**

Air leakage and hemorrhage are important causes of morbidity and mortality in operations and traumas of the lung. Ankaferd Blood Stopper is a herbal product used for stopping hemorrhage. In our study, we investigated the efficacy of Ankaferd Blood Stopper in the prevention of air leakage in the lung and bleeding.

**Methods:**

A total of twenty-one Wistar-Albino rats weighing 240 ± 20 grams were used in our study. An equal amount of injury was created in all groups by performing left thoracotomies. No interventions were made on tissue injury in the first group, and suturing was performed in the second group, and Ankaferd was applied in the third group. Air leakage and duration of bleeding were recorded in all groups.

**Results:**

A statistically significant difference was found between the three groups in terms of air leakage time (p = 0,0001) and bleeding time (p = 0,0001). While a significant effect of Ankaferd was detected in terms of air leakage compared to standard surgery (p = 0,017), no difference was found in terms of bleeding time.

**Conclusions:**

Ankaferd Blood Stopper ceases the air leakage in the lung parenchyma significantly and effectively. No significant difference is seen compared to the standard surgery group, although it ceases bleeding significantly.

## Introduction

Prolongation of air leakage and bleeding after lung operations are among the important causes of morbidity. Prolongation of air leakage is the second leading cause of delay in the time of discharge from the hospital [1]. Prolonged parenchymal air leakage is commonly seen after lung resections and has been reported at a rate of 15-18%. Thus, chest tubes are needed for a longer time and consequently, this causes pain, decreased mobility and possible complications [2]. The standard method for prevention of air leakage and bleeding developing following lung resection is surgical suturing or stapler application. Tissue adhesives and supported stapler use are the other methods [3-10].

Ankaferd Blood Stopper (ABS) is a herbal product used as a hemostatic agent in traditional Turkish medicine. It is a topical agent, the safety and efficacy of which have been proven in dermal, external traumatic, postoperative and dental bleedings [8]. Clinical and experimental studies on its blood stopping effect are available. Its effect on stopping bleeding and preventing air leakage in the lungs is not known. In this study, we experimentally investigated the effect of Ankaferd Blood Stopper on stopping bleeding and preventing air leakage in the lungs.

## Methods

This study was carried out in the animal experiment laboratory of our institution. All rats were treated in a humane manner according to the Guide for the Care and Use of Laboratory Animals. The study was commenced after having obtained approval from the ethical committee for experimental animals.

A total of 21 adult male Wistar Albino rats weighing 240 ± 20 grams were used in our study. Three groups were constituted with seven rats in each. An equal extent of injury was created in all groups by performing left thoracotomies. No interventions were made on the tissue injury in the first group (control group), suturing was performed in the second group (standard surgery group), and Ankaferd Blood Stopper was applied in the third group (Ankaferd group). Air leakage and duration of bleeding were recorded in all groups. The rats were sacrificed after the procedure had been completed and histopathological assessment was performed.

### Anesthesia Technique

General anesthesia was applied on all test subjects. A combination of 60 mg/kg ketamin hydrochloride (Ketalar, Parke Davis-Eczacıbası, İstanbul, Turkey) and 10 mg/kg xylacine hydrochloride (Rompun, Bayer, Toronto, Canada) were used as anesthetic agents. The rats were fixed in the supine position. The operation sites were cleaned and sterilization was provided. Tracheotomies were performed with neck incisions and intubation was performed.

### Surgical Technique

All the rats underwent left anterior thoracotomy. An injury 5 mm in length and 2mm in depth was created in the left lung parenchyma using a scalpel. In the control group (Group I), no interventions were made for parenchymal injury and a compress was applied and continuation of the air leakage and bleeding was monitored every 5 seconds. These durations were recorded. The procedure was terminated at the 100. second. In the standard surgery group (Group II), after having created an equal injury, the injury was sutured using 6-0 polyglactin (vicryl). Then the air leakage and bleeding were controlled and the times of cessation of the leakage and bleeding were recorded. In the Ankaferd group (Group III), a spray form of the Ankaferd Blood Stopper was applied 4 times onto the identical injury. It was controlled at every 5 seconds and cessation times of the air leakage and bleeding were recorded.

### Statistical Analysis

All statistical analyses and calculations were performed using the SPSS for Windows Version 16.0 (SPSS Inc, Chicogo, IL, USA) package program. The Kruskal Wallis test was used to find whether or not there was a difference between the three groups in terms of air leakage time and bleeding time. Paired assessments were made using the Mann Whitney U test to find the group where the difference originated from. The level of significance was set at p < 0.05.

### Histopathological Analysis

Some of the rats (n = 11) were sacrificed intraoperatively after the procedure had been completed and the specimens were obtained from the lung. The remaining rats (n = 10) were sacrificed after monitorization for five days and the lung was analyzed patologically on the fifth day. The sampled lung tissue was fixed with formalin solution (10%). Paraffin cross sections were obtained after routine follow-up and analyzed after staining with hematoxylin-eosin.

## Results

**Figure 1:**
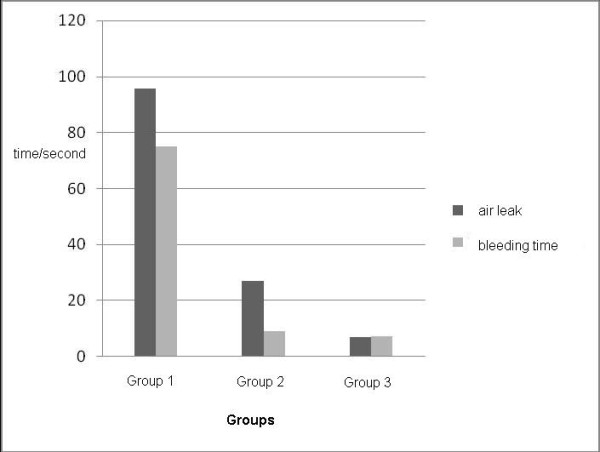
**Distribution of air leakage and bleeding times in the control group (Group 1, n = 7), standard surgery group (Group 2, n = 7) and Ankaferd Blood Stopper group (Group 3, n = 7)**.

While the mean air leakage time was 95.7 ± 6.07 sec, the bleeding time was measured as 75.00 ± 15.00 sec in the control group. While the mean time of air leakage was 27.14 ± 21.76 sec, the bleeding time was measured as 9.28 ± 7.31 sec in the standard surgery group, whereas both the air leakage time and the bleeding time were measured as 7.14 ± 2.68 sec in the Ankaferd group (Table 1) (Figure 1). Bleeding and air leakage were found to have stopped in the shortest mean duration in the Ankaferd group.

**Table 1:** Mean air leakage and bleeding times of the groups

Groups	Mean air leakage time (sec)	Mean bleeding time (sec)
Group I (control group)	95.7 ± 6.07	75.00 ± 15.00
Group II (standard surgery group)	27.14 ± 21.76	9.28 ± 7.31
Group III (Ankaferd group)	7.14 ± 2.67	7.14 ± 2.68

A statistically significant difference was found between the three groups in terms of duration of air leakage (p = 0,0001) and bleeding (p = 0,001). There was a statistically significant difference between the control group and the standard surgery group in terms of duration of leakage (p = 0,002) and bleeding time (p = 0,001). A statistically significant difference was found between the standard surgery group and the Ankaferd group in terms of air leakage (p = 0,017). No statistically significant difference was found between the standard surgery group and the Ankaferd group in terms of bleeding time (p = 0,827). A statistically significant difference was detected between the control group and the Ankaferd group in terms of air leakage time (p = 0,001) and bleeding time (p = 0,001).

**Figure 2:**
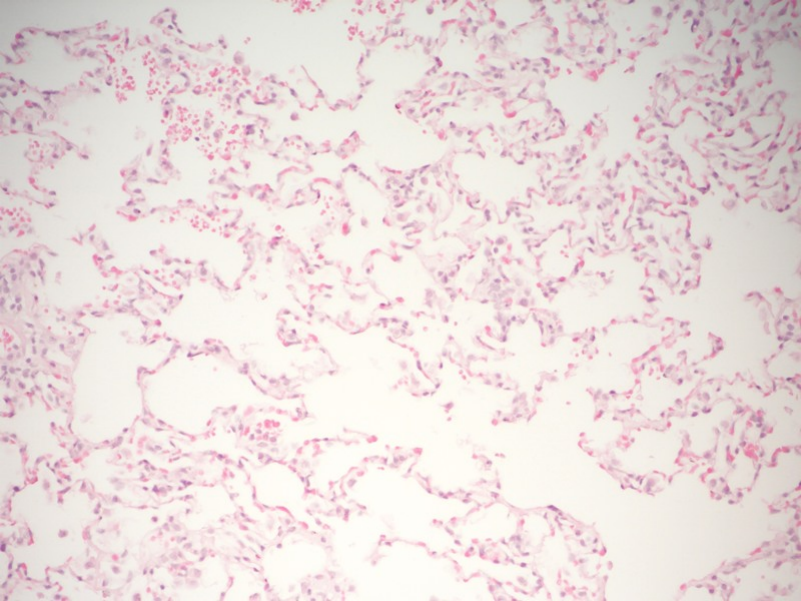
**Appearance of lung tissue of the normal rats ( H&E, X200)**.

**Figure 3:**
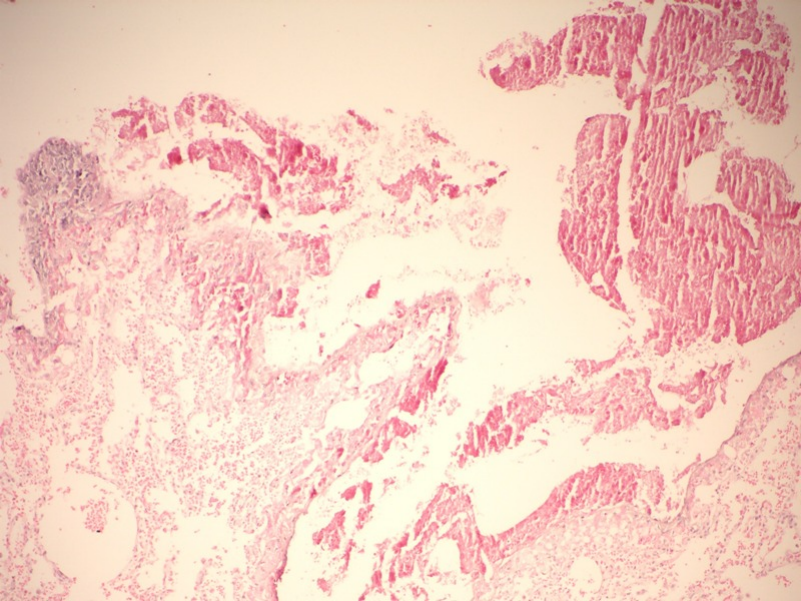
**Changes in the early period (intraoperative) in the lung in which Ankaferd was applied**. Hemolyzed clot masses (blood-fibrin masses) are observed in the areas exposed to Ankaferd ( H&E, X200)

**Figure 4:**
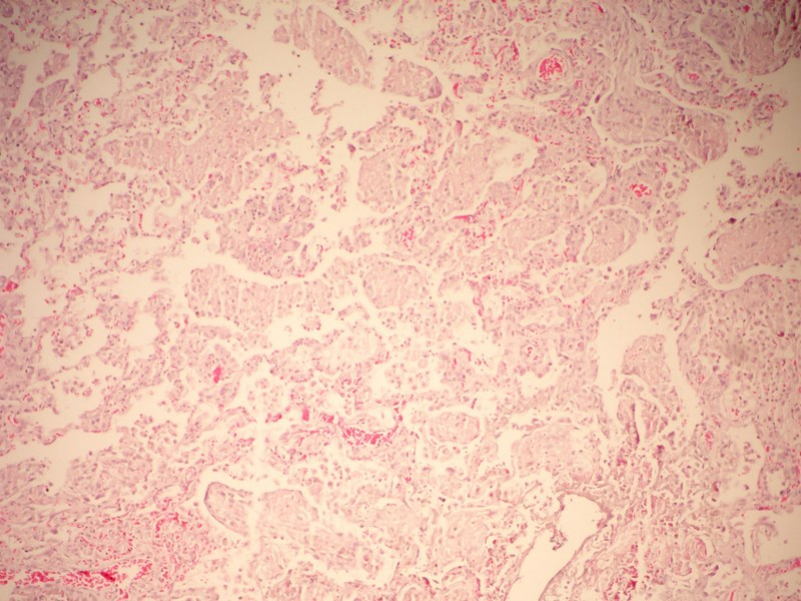
**Histopathological changes on the 5. day in the lung in which Ankaferd was applied**. Newly organized fibrin plugs are observed in the alveoli of the lung of Ankaferd-applied rats( H&E, X200)

Normal lung regions (Figure 2) and the lungs of rats sacrificed intraoperatively and on the fifth day were analyzed in the histopathological evaluation. Masses of hemolyzed clot (blood-fibrin masses) were observed in the areas in which Ankaferd had been applied in the lungs of intraoperatively sacrificed rats (Figure 3). Newly organized fibrin plugs were observed in the alveoli of the lungs of rats that had been sacrificed on the fifth, in which Ankaferd had been applied (Figure 4).

## Discussion

The incidence of leakage from the lung parenchyma is still vey high despite the use of many surgical techniques and biological agents to reduce it. Leakage is observed at a rate between 48% and 70% intraoperatively, and the rate of air leakages continuing for longer than the 7. postoperative day is between 15% and 18%. The ideal method for preventing this has yet not been determined [2].

Prolongation of air leakage is the second leading cause of delay in the time of discharge from the hospital following pain [1]. Prolongation of air leakage following lung resections is among the important causes of morbidity. It is the second leading complication following arrhythmias. Cause of morbidity is air leakage exceeding five days after segmentectomy (5.9%), lobectomy (9.6%) and pneumectomy (0.4%) [11]. In the study of Varela et al. [12] air leakage exceeding five days was found to be related to significant pulmonary complications and atelectasis, pneumonia, and empyema.

Air lekage is a common condition seen after lung operations. The standard method for prevention of air leakages developing following lung operations is surgical suturing or stapler application. The other methods are fibrin glue, synthetic polyethylene glucose-based hydrogel adhesives, tachocomb, covered adhesives or stapler use supported with various materials [3-10].

Fibrin glue application is a commonly used approach. There are different opinions about the utilization of fibrin glue. In the prospective study carried out by Fabian et al. [4] fibrin glue was applied to one group in 100 lung resections. Both groups were compared in terms of air leakage, amount of pleural drainage, time of chest tube removal and duration of hospitalization. Utilization of fibrin glue was shown to significantly decrease the incidence of air leakage, the air leakage cessation time, chest tube removal time, and the rate of prolonged air leakage. No difference was found in terms of the amount of chest tube drainage and duration of hospitalization. No complications related to fibrin glue were found. In conclusion, fibrin glue utilization was reported to be effective and safe. In a study carried out with fibrin glue comprising 360 patients, it was shown to decrease only the chest tube removal time and not to affect the prolongation of air leakage (>7 days) and duration of hospitalization [5].

Ankaferd Blood Stopper (ABS) is a herbal product used as a hemostatic agent. It contains a standardized mixture of Thymus vulgaris, Glycyrrhiza glabra, Vitis vinifera, Alpinia officinarum and Urtica dioica. All of these plants are effective on the endothelium, blood cells, angiogenesis, cellular proliferation, vascular dynamics and mediators. The mechanism of action of this drug which is being used clinically and found to be safe is not fully understood [13]. There have been no studies investigating the effect of Ankaferd Blood Stopper on air leakage in the lung. In our study, we investigated the air leakage repressive effect of ABS. It prevents air leakage significantly compared to the control group and the standard surgery group.

Postoperative hemorrhage is among the important causes of morbidity in thoracic surgery practice. The incidence of at least 4 units of blood transfusion requirement after lung resections is 2.9% in lobectomy, 3% in pneumonectomy [11]. The focus of hemorrhage cannot be determined in most of the cases. In the study of Sirbu et al. analyzing 1960 patients who underwent thoracotomy, they detected that the most common cause of re-thoracotomy was bleeding (52%). In this study, while the source of bleeding was found to be mediastinal and bronchial vessels (23%), intercostal vessels (17%) or pulmonary vessels (17%), no sources of bleeding could be detected in most of the cases (41%) [14].

The blood stopping effect of Ankaferd Blood Stopper has been demonstrated in many clinical and experimental studies. It was found to be successful in the treatment of rectal ulcers in the study of Ibis et al. [15] which was carried out in gastrointestinal hemorrhages. It was shown to reduce hemorrhage considerably in bladder hemorrhages and partial nephrectomies [16]. Ankaferd Blood Stopper was shown to decrease the operation time and warm ischemia time in the partial nephrectomy model in rats. In the pathological anaysis, erythrocyte aggregation was found to develop, but glomerular necrosis and calcifications were not observed [16]. ABS was reported to reduce hemorrhage effectively in the study on its clinical use in tonsillectomies [17]. It was emphasized that it could be beneficial clinically when used endoscopically in gastrointestinal hemorrhages related to tumor [18]. Similarly, it was stated to be effective in hemorrhages related to endobronchial tumor when used endoscopically [19].

In our study, when we evaluated the blood stopping effect of ABS, we found that this property was prominent compared to the control group; however, there was no statistically significant difference compared to the standard surgery group. Ankaferd Blood Stopper prevents air leakage in the lung effectively. It has an effect on stopping bleeding, but the effect is similar to that in standard surgery. Further experimental and clinical studies are needed to investigate the effect of this plant extract in the lung.

## Competing interests

The authors declare that they have no competing interests.

## Authors' contributions

AK, NGS, TK and GB conceived of the study, and participated in its design and coordination and helped to draft and performed the statistical analysis. ÇB carried out the macroscopic and microscopic studies. ÖS, AK, and TK participated in the design of the study. AK and ÖS participated in the sequence alignment and drafted the manuscript. All authors read and approved the final manuscript.
